# Effect of hypoxia and Beraprost sodium on human pulmonary arterial smooth muscle cell proliferation: the role of p27^kip1^

**DOI:** 10.1186/1465-9921-8-77

**Published:** 2007-11-01

**Authors:** Maiko Kadowaki, Shiro Mizuno, Yoshiki Demura, Shingo Ameshima, Isamu Miyamori, Takeshi Ishizaki

**Affiliations:** 1 Third Department of Internal Medicine, University of Fukui, 23-3 Eiheiji-cho, Matsuoka, Yoshida-gun, Fukui, Japan; 2 Department of Fundamental Nursing, University of Fukui, 23-3 Eiheiji-cho, Matsuoka, Yoshida-gun, Fukui, Japan

## Abstract

**Background:**

Hypoxia induces the proliferation of pulmonary arterial smooth muscle cell (PASMC) *in vivo *and *in vitro*, and prostacyclin analogues are thought to inhibit the growth of PASMC. Previous studies suggest that p27^kip1^, a kind of cyclin-dependent kinase inhibitor, play an important role in the smooth muscle cell proliferation. However, the mechanism of hypoxia and the subcellular interactions between p27^kip1 ^and prostacyclin analogues in human pulmonary arterial smooth muscle cell (HPASMC) are not fully understood.

**Methods:**

We investigated the role of p27^kip1 ^in the ability of Beraprost sodium (BPS; a stable prostacyclin analogue) to inhibit the proliferation of HPASMC during hypoxia. To clarify the biological effects of hypoxic air exposure and BPS on HPASMC, the cells were cultured in a hypoxic chamber under various oxygen concentrations (0.1–21%). Thereafter, DNA synthesis was measured as bromodeoxyuridine (BrdU) incorporation, the cell cycle was analyzed by flow cytometry with propidium iodide staining. The p27^kip1 ^mRNA and protein expression and it's stability was measured by real-time RT-PCR and Western blotting. Further, we assessed the role of p27^kip1 ^in HPASMC proliferation using p27^kip1 ^gene knockdown using small interfering RNA (siRNA) transfection.

**Results:**

Although severe hypoxia (0.1% oxygen) suppressed the proliferation of serum-stimulated HPASMC, moderate hypoxia (2% oxygen) enhanced proliferation in accordance with enhanced p27^kip1 ^protein degradation, whereas BPS suppressed HPASMC proliferation under both hypoxic and normoxic conditions by suppressing p27^kip1 ^degradation with intracellular cAMP-elevation. The 8-bromo-cyclic adenosine monophosphate (8-Br-cAMP), a cAMP analogue, had similar action as BPS in the regulation of p27^kip1^. Moderate hypoxia did not affect the stability of p27^kip1 ^protein expression, but PDGF, known as major hypoxia-induced growth factors, significantly decreased p27^kip1 ^protein stability. We also demonstrated that BPS and 8-Br-cAMP suppressed HPASMC proliferation under both hypoxic and normoxic conditions by blocking p27^kip1 ^mRNA degradation. Furthermore, p27^kip1 ^gene silencing partially attenuated the effects of BPS and partially restored hypoxia-induced proliferation.

**Conclusion:**

Our study suggests that moderate hypoxia induces HPASMC proliferation, which is partially dependent of p27^kip1 ^down-regulation probably *via *the induction of growth factors such as PDGF, and BPS inhibits both the cell proliferation and p27^kip1 ^mRNA degradation through cAMP pathway.

## Background

Exposure to chronic hypoxia leads to pulmonary hypertension (PH) associated with the structural remodeling of pulmonary vessels [1-3]. Many pulmonary disorders are associated with chronic hypoxia, accompanied by pulmonary hypertension and fatal right heart failure resulting from pulmonary vascular remodeling [4-6]. Prolonged exposure to hypoxia is associated with cellular and histological changes in vascular remodeling, and the key pathological findings of pulmonary vascular remodeling are increased wall thickening of pulmonary vessels and the muscularization of small arteries. Decreased ambient oxygen concentrations in laboratory animals cause similar pathological changes, including pulmonary smooth muscle hypertrophy and proliferation [7,8]. Furthermore, several studies *in vitro *have also shown that exposure to hypoxia stimulates pulmonary arterial smooth muscle cell (PASMC) proliferation, which might be a key component of pulmonary vascular remodeling [9-12].

Prostacyclin (PGI_2_) is thought to improve exercise tolerance and survival in patients with either primary or secondary PH through its ability to inhibit the growth of PASMC [13-15]. TORAY Industries Inc. developed Beraprost sodium (BPS), which was the first chemically stable and orally active PGI_2 _analogue to increase intracellular cAMP levels via adenylate cyclase activation [16]. Since 1995, BPS has been used to treat PH and obstructive peripheral arterial disease [17,18]. The drug mimics the biological properties of PGI_2_, such as activating adenylate cyclase and increasing intracellular cAMP levels, through activation of the PGI_2 _receptor. Owing to its chemical characteristics, BPS is more stable and persistent than natural PGI_2 _and has higher affinity for the PGI_2 _receptor [19].

The proliferation of PASMC, which causes pulmonary vascular remodeling, requires the cells to enter the cell cycle. The most important molecular process for cell cycle progression is retinoblastoma protein phosphorylation by cyclin-dependent kinase (CDK)-cyclin complexes, and CDK activities are mainly regulated by CDK inhibitors [20] such as p27^kip1^. Other studies have found that the CDK inhibitor, p27^kip1^, plays an important role in the inhibition of CDK activity and in the proliferation of vascular smooth muscle cells [8,21-23]. On the other hand, Li et al. found that BPS suppresses systemic vascular smooth muscle proliferation through cAMP signaling via p27^kip1 ^expression [24]. On the contrary, cell cycle arrest at late G_1 _is caused by p27^kip1 ^expression under severe hypoxia [25-27]. These results support the notion that the oxygen-dependent checkpoint of the cell cycle is controlled by p27^kip1 ^expression, and that cAMP signaling also interferes with the cell cycle and p27^kip1 ^expression. However, the precise mechanisms and interactions between the pathways activated by hypoxia, as well as the antiproliferative effects of p27^kip1 ^during exposure to BPS in pulmonary arterial smooth muscle cells remain uncertain. We aimed to clarify the inhibitory effect of BPS in cultured human pulmonary arterial smooth muscle cells (HPASMC), as well as interactions of the CDK inhibitor p27^kip1^. We assessed the effects of BPS and 8-bromo-cyclic adenosine monophosphate (8-Br-cAMP), a cAMP analogue, on cell proliferation and p27^kip1 ^expression, and examined the role of p27^kip1 ^in HPASMC proliferation using p27^kip1 ^gene silencing.

## Methods

### Reagents

We obtained reagents and materials from various sources as follows: Humedia SG medium, recombinant human EGF and FGF, gentamycin, streptomycin, and amphotericin B (Kurabo Ltd., Osaka, Japan); bromodeoxyuridine (BrdU) proliferation assay kits (Oncogene™, Cambridge, MA); low-pH cAMP ELISA kits (R&D Systems, Inc., Minneapolis, MN); ECL detection system (Amersham, Buckinghamshire, UK); Moloney murine leukemia virus reverse transcriptase (Toyobo Co. Ltd., Osaka, Japan), Quantitech™ SYBR Green PCR kits (Qiagen, Santa Clarita, CA); Lipofectamine 2000, 4 – 12% Bis-Tris Nupage gels, and MES-SDS running buffer (Invitrogen, Carlsbad, CA); DC protein assay kit and polyvinylidene difluoride (PVDF) membranes (Bio-Rad Laboratories, Richmond, CA; rabbit anti-p27^kip1 ^polyclonal antibody, mouse anti-β-actin monoclonal antibody, horseradish peroxidase-conjugated goat anti-mouse and rabbit antibody, p27^kip1 ^and control small interfering RNA (siRNA) (Santa Cruz Biotechnology Inc., Santa Cruz, CA) and BPS was a gift from Toray Industries Inc. (Tokyo, Japan). All other chemicals were purchased from Sigma (St. Louis, MO).

### Cell culture

HPASMC supplied by Kurabo Ltd. (Osaka, Japan) were cultured in Humedia SG medium containing 5% fetal bovine serum, with 50 μg/ml of gentamycin, 50 ng/ml of amphotericin B, 1 ng/ml of recombinant human EGF, and 1 ng/ml of recombinant human FGF. The cells were incubated in 75-cm^2 ^tissue culture flasks (Corning, NY, U.S.) in a cell-culture incubator (37°C, 5% CO_2_, and 95% air) and used at the seventh passage after trypsinization in all experiments. Oxygen concentrations (0.1% ~10%) were modified using N_2_-CO_2 _incubators (BNR-110M; Tabai ESPEC Corp., Tokyo, Japan; 10-0233, Ikemoto Rika Kogyo, Co., LTD., Tokyo, Japan).

### Incorporation of BrdU into HPASMC

We incubated HPASMC (6,000 cells/cm^2^) seeded in 96-well culture plates for 48 h in serum-free DMEM, then changed the medium to DMEM containing 10% FBS and antibiotics. Thereafter, the cells were incubated for 24 h in various oxygen concentrations with or without 10 μM BPS. We measured BrdU incorporation using BrdU proliferation assay kits according to the manufacturer's protocol. Briefly, the cells were labeled with 10 ng/ml of BrdU during the incubation, washed 3 times with cold PBS, fixed, air dried and incubated with mouse anti-BrdU monoclonal antibody (diluted 1:1,000). The antibody was aspirated, the cells were washed 3 times and then incubated with peroxidase goat anti-mouse IgG (1:2,000) at room temperature for 30 minutes. The cells were washed 3 times, and 100 μM substrate was added to each well and incubated for 10 minutes in darkness. Thereafter, we measured absorbance at dual-wave lengths of 450 to 540 nm.

### Cell cycle and DNA analyses

We examined whether the cell cycle was influenced by the oxygen concentration using flow cytometry with propidium iodide staining. We incubated HPASMC (6,000 cells/cm^2^) seeded in 6-well culture plates for 48 h in serum free DMEM, then changed the medium to DMEM containing 10% FBS and antibiotics. The cells were further incubated for 24 h under normal or hypoxic conditions with or without 10 μM BPS. The cells were harvested with trypsin-EDTA and fixed using 70% ethanol. The ethanol was removed and the cells were incubated in PBS containing RNase (172 k units/ml) at 37°C for 30 minutes, stained with propidium iodide (50 μg/ml) and suspended in PBS for 30 minutes on ice. DNA fluorescence was measured and flow cytometry proceeded using an EPICS XL (Beckman Coulter, CA).

### Assay of intracellular cAMP expression in HPASMC

We incubated HPASMC (6,000 cells/cm^2^) seeded in 24-well culture plates for 48 h in serum-free DMEM then changed the medium to DMEM containing 10% FBS. The plates were incubated for various periods under normal or hypoxic conditions in the presence of 10 μM BPS. The medium was aspirated and adherent cells were solubilized with 200 μl of 0.1 N HCl and 0.1% Triton X. Thereafter, cAMP concentrations in the cell lysates were measured using a low-pH cAMP ELISA kit according to the manufacturer's protocol.

### Real-time RT-PCR analysis of p27^kip1 ^using LightCycler™

We cultured HPASMC (6,000 cells/cm^2^) seeded in 6-cm dishes for 48 h in serum-free DMEM. The cells were washed twice with PBS, and then placed in DMEM containing 10% FBS and antibiotics under normal or hypoxic oxygen concentrations for various periods with or without 10 μM BPS or 1 mM of 8-Br-cAMP. The cells were then harvested by trypsinization, washed 3 times, and pelleted by centrifugation. Total cellular RNA was obtained by one acid guanidinium thiocyanate-phenol-chloroform extraction [28]. Reverse transcription proceeded using 0.5 μg of total RNA and cDNA was synthesized using 200 U of Moloney murine leukemia virus reverse transcriptase, 5 μM oligoDT, 1 mM dNTPs, and 3 mM Mg^2+ ^in a total volume of 20 μl. Annealing proceeded at room temperature for 5 minutes, extension at 44°C for 40 minutes, and chain termination at 99°C for 5 minutes.

We then performed PCR using the RT products and specific oligonucleotide primers for p27^kip1 ^and β-actin. The sequences of the forward and reverse primers for p27^kip1 ^were 5'-GCCCTCCCCAGTCTCTCTTA-3' and 5'-TCAAAACTCCCAAGCACCTC-3', respectively, and those of the forward and reverse primers for β-actin were 5'-GCAAGCAGGAGTATGACGAG-3' 5'-CAAATAAAGCCATGCCAATC-3', respectively. All PCR reactions proceeded using a LightCycler™ PCR system (Roche Diagnostics, Meylan, France) using DNA-binding SYBR green dye to detect PCR products. The cycling conditions were as follows: initial denaturation at 95°C for 15 minutes, 50 cycles of denaturation at 94°C for 15 seconds, annealing at 55°C for 15 seconds, and extension at 72°C for 15 seconds. The β-actin gene served as the reference. The PCR products were isolated from the LightCycler™ glass capillaries, resolved by electrophoresis on 1.5% agarose gels and confirmed by ethidium bromide (EB) staining. Each assay was repeated in 6 independent experiments.

### Western blot analysis

We cultured HPASMC (6,000 cells/cm^2^) seeded in 6-cm dishes for 48 h in serum-free DMEM. The cells were washed twice with PBS, placed in DMEM containing 10% FBS and antibiotics and then cultured under normal or hypoxic oxygen conditions for various periods with or without 10 μM BPS or 1 mM 8-Br-cAMP. The cells were then harvested and resuspended in protein lysis buffer (150 mM of NaCl, 20 mM of Tris-HCl, 1% NP-40, 10 mM of EDTA, 10% glycerol, 1 mM of PMSF, 10 μg/ml of aprotinin, 1 μg/ml of leupeptin, 1 μg/ml of pepstatin) and incubated for 30 min on ice. Cell lysates were clarified by centrifugation at 10,000 g for 15 minutes at 4°C, then the protein content in the supernatants was quantified using DC protein assay kits. Thereafter, 25 μg of protein per lane was loaded onto 4 – 12% Bis-Tris Nupage gels with MES SDS running buffer, according to the manufacturer's protocol. The gels were transferred to PVDF membranes by electrophoresis at 100 V for 1 h, then non-specific binding was blocked in PBS containing 0.2% Tween 20 (PBS-T) and 5% nonfat milk (blocking buffer) at room temperature for 1 h. All antibodies were diluted in blocking buffer. The membrane was then probed with rabbit anti-p27^kip1 ^polyclonal antibody (diluted 1:1,000) or mouse anti-β-actin monoclonal antibody (diluted 1:5000), and incubated for 1 h at room temperature. Membranes were washed with PBS-T and incubated with horseradish peroxidase-conjugated goat anti-rabbit or mouse IgG (diluted 1:2,000) for 2 h at room temperature. After washing with PBS-T, proteins were detected using the ECL system. Each assay was repeated in 4 independent experiments.

### Analysis of p27^kip1 ^mRNA and protein stability

We cultured HPASMC (6,000 cells/cm^2^) seeded in 6-cm dishes for 48 h in serum-free DMEM. The cells were washed twice with PBS, then placed in DMEM containing 10% FBS and cultured under normal or hypoxic conditions for the indicated periods in the presence of the transcription inhibitor actinomycin D (Act D) (400 nM), or the protein synthesis inhibitor cycloheximide (CHX) (25 μg/ml), and with or without 10 μM BPS, 1 mM 8-Br-cAMP or 25 ng/ml of platelet-derived growth factor (PDGF). The cells were then counted and mRNA and protein stability was examined per 50,000 cells incubated with Act D and CHX using RT-PCR and Western blotting, respectively. Each assay was repeated in 4 independent experiments.

### Transfection of siRNA in HPASMC

We incubated HPASMC in 10-cm dishes in DMEM containing 10% FBS for 24 h, until they reached about 60% confluence. After rinsing, the cells were incubated for 6 h with serum-free Opti-MEM medium, 5 μl/ml of Lipofectamine 2000, and 50 nM control or p27^kip1 ^siRNA. The same amount of Opti-MEM medium containing 20% FBS was added and the cells were incubated for a further for 16 h. At 24 h after transfection, the cells were cultured in serum-free DMEM for 48 h, harvested and seeded (6,000 cells/cm^2^) into 6-cm dishes and 96-well culture plates, then incubated in DMEM supplemented with 10% FBS and antibiotics for 24 h under normal or hypoxic conditions with or without 10 μM BPS. We then measured BrdU incorporation into the transfected cells and confirmed target gene silencing by p27^kip1 ^siRNA using Western blotting.

### Statistical analysis

The results are expressed as means ± SE. Statistical analysis was performed using ANOVA with Bonferroni correction for multiple comparisons. Comparisons were considered statistically significant at p < 0.05.

## Results

### Effects of BPS on HPASMC proliferation during hypoxia

**Figure 1:**
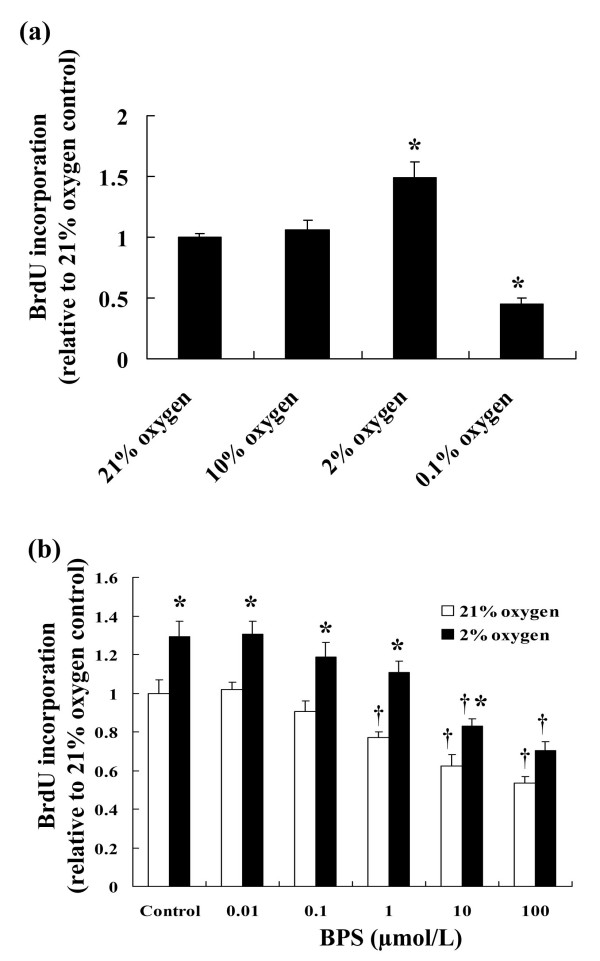
**Effects of hypoxia and BPS on BrdU incorporation in cultured HPASMC**. Cultured HPASMC were exposed to various concentrations of oxygen and BPS in the presence of BrdU for 24 hours. (a) Severe hypoxia (0.1% oxygen) suppressed, whereas moderate hypoxia (2% oxygen) significantly enhanced BrdU incorporation. *P < 0.05 versus 21% oxygen. (b) BrdU incorporation was dose-dependently suppressed by BPS under both normoxic and hypoxic conditions. Data are expressed as means ± SE (n = 6). Open bars, 21% oxygen; solid bars, 2% oxygen. *P < 0.05 versus 21% oxygen control; ^†^P < 0.05 versus without BPS.

Moderate hypoxia (2% oxygen) promoted, whereas severe hypoxia (0.1% oxygen) suppressed DNA synthesis in serum-stimulated HPASMC (Fig. 1a). Under normal and moderately hypoxic conditions, BPS dose-dependently suppressed DNA synthesis starting at concentrations of 1 and 10 μM, respectively (Fig. 1b).

**Figure 2:**
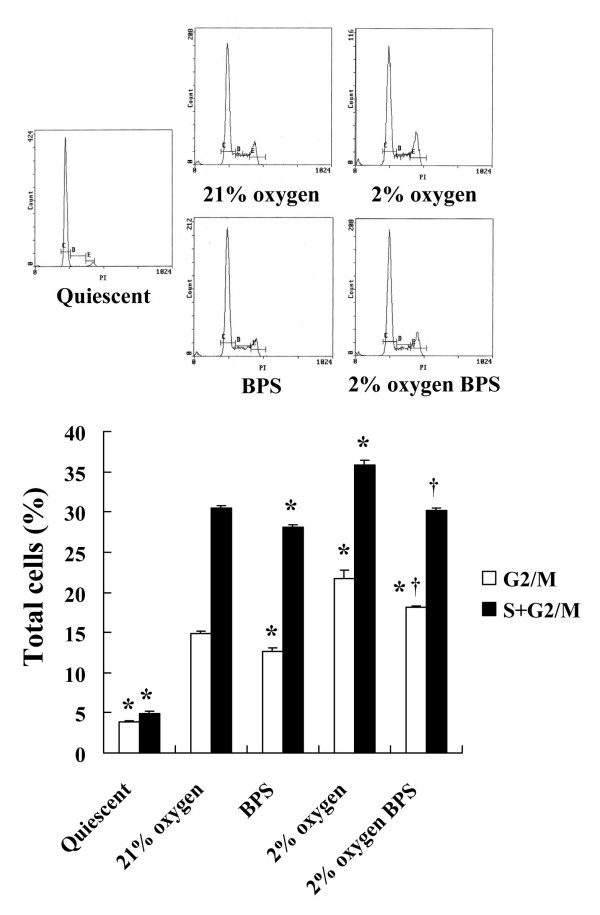
**Cell cycle analysis of HPASMC exposed to hypoxia and BPS**. Cultured HPASMC were exposed to 21% and 2% oxygen with or without 10 μM BPS for 24 hours. Cells were harvested and DNA fragmentation was analyzed using flow cytometry and propidium iodide staining. Area definitions on DNA histograms: C, G_0/1 _phase; D, S phase; E, G_2_/M phase. Moderate hypoxia (2% oxygen) increased, whereas BPS significantly decreased ratios of S plus G_2_/M and G_2_/M phases. Histograms are representative and bar graph shows data expressed as means ± SE (n = 4). Open bars, ratios of G_2_/M phases; solid bars, ratios of S plus G_2_/M phases. *P < 0.05 versus 21% oxygen control; ^†^P < 0.05 versus 2% oxygen without BPS.

Cell cycle visualization by PI staining showed that about 95% of the cells cultured in serum-free DMEM was synchronized at the G_0/1 _phase and that the cell cycle was arrested (quiescent state). Moderate hypoxia significantly promoted cell cycle progression and forced the cells to enter the S and G_2_/M phases compared with the control under normal oxygen conditions and BPS significantly suppressed the cell cycle progression of cells that were serum-stimulated under hypoxic conditions (Fig. 2).

### Effects of BPS on cAMP production during hypoxia

**Figure 3:**
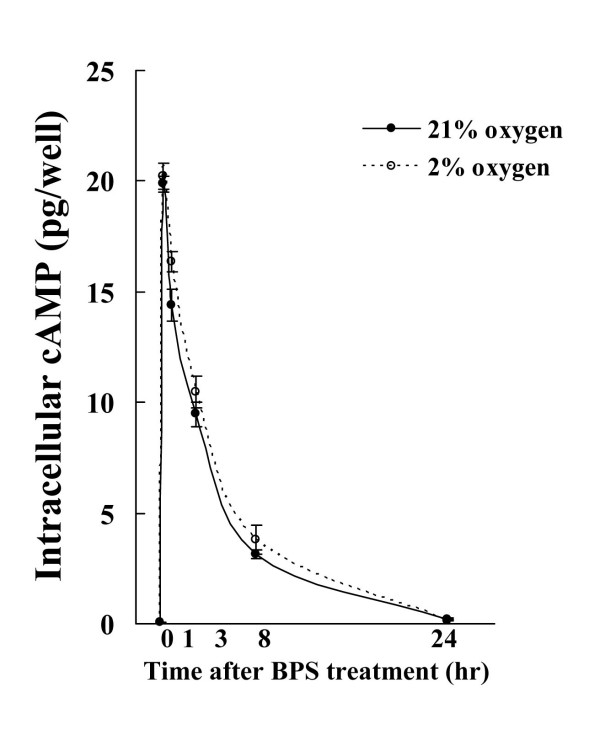
**Effects of BPS on intracellular cAMP production during hypoxia**. Cultured HPASMC were exposed to 21% or 2% oxygen in the presence or absence of 10 μM of BPS for indicated periods. Concentrations of cAMP in cell lysates were measured using low-pH cAMP ELISA kits. Although BPS significantly induced cAMP expression, intracellular cAMP expression did not significantly differ between the indicated oxygen concentrations. Line with solid circles, 21% oxygen; dotted line with open circles, 2% oxygen. Data are expressed as means ± SE (n = 6).

Intracellular cAMP production was elevated by 10 μM BPS from 15 min until 24 h. Intracellular cAMP production did not significantly differ between ambient and hypoxic oxygen concentrations (Fig. 3).

### Effects of BPS and 8-Br-cAMPon p27^kip1 ^mRNA expression during hypoxia

**Figure 4:**
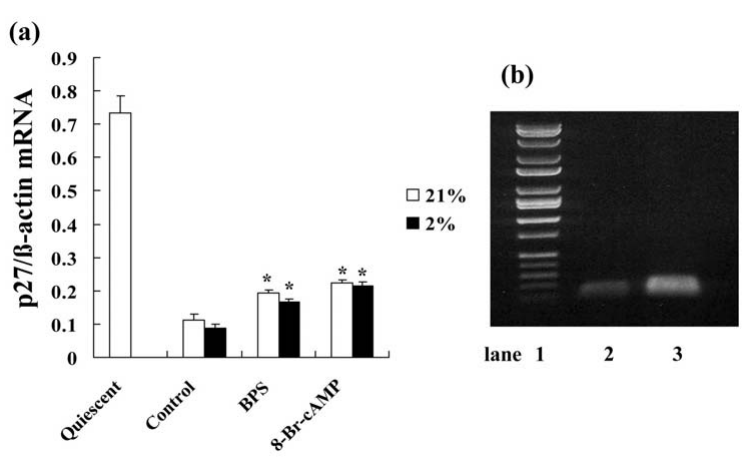
**Effects of BPS and 8-Br-cAMPon p27^kip1 ^mRNA expression during hypoxia**. Cultured HPASMC were exposed to 21% or 2% oxygen concentrations with or without 10 μM of BPS or 1 mM 8-Br-cAMP for indicated periods. Expression of p27^kip1 ^mRNA was measured using Real-time RT-PCR using LightCycler™. (a) BPS suppressed p27^kip1 ^mRNA reduction under both normoxic and hypoxic conditions. Expression of p27^kip1 ^mRNA between normoxic and hypoxic conditions did not significantly change. Graph shows ratio of p27^kip1 ^to β-actin mRNA expression. Open and solid bars, 21% and 2% oxygen, respectively. Data are expressed as means ± SE (n = 6). *P < 0.05 versus control. (b) Agarose gel electrophoresis with EB staining revealed single amplification of predicted PCR products (lane 1, DNA molecular weight markers; lane 2, p27^kip1^; 109 bp; lane 3, β-actin 144 bp).

After incubation in serum-depleted medium (quiescent state), p27^kip1 ^mRNA expression was obviously up-regulated, and decreased by 24 h of serum stimulation. On the other hand, BPS and 8-Br-cAMP significantly attenuated the suppression induced by 24 h of serum stimulation. However, p27^kip1 ^mRNA expression did not significantly differ between normoxia and moderate hypoxia (Fig. 4a).

**Figure 5:**
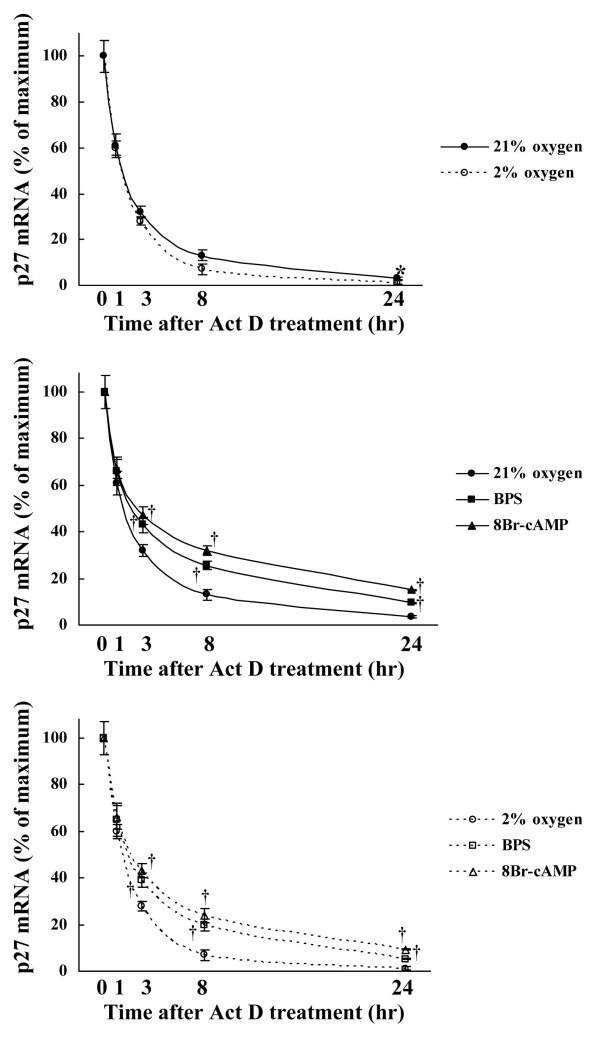
**Effect of BPS and 8-Br-cAMP on p27^kip1 ^mRNA stability during hypoxia**. Cultured HPASMC were exposed to 21% or 2% oxygen concentrations with or without 10 μM of BPS or 1 mM 8-Br-cAMP for indicated periods. The p27^kip1 ^mRNA stability was measured after adding 400 nM of Act D using Real-time RT-PCR using LightCycler™. Degradation of p27^kip1 ^mRNA was significantly suppressed by BPS and 8-Br-cAMP under both normoxic and moderately hypoxic conditions, and mRNA stability was slightly decreased by moderate hypoxia. Graphs show % maximal p27^kip1 ^mRNA expression. Line with solid circles, 21% oxygen; dotted line with open circles, 2% oxygen; line with solid squares, 21% oxygen and BPS; line with solid triangles, 21% oxygen and 8-Br-cAMP; dotted line with open squares, 2% oxygen and BPS; dotted line with open triangle, 2% oxygen and 8-Br-cAMP. Data are expressed as means ± SE (n = 6). *P < 0.05 versus 21% oxygen. ^†^P < 0.05 versus oxygen controls.

To confirm the effect of BPS and hypoxia on p27^kip1 ^mRNA expression, we assessed p27^kip1 ^mRNA stability using Act D. Both BPS and 8-Br-cAMP significantly suppressed p27^kip1 ^mRNA degradation in cells incubated with Act D under both normoxic and moderately hypoxic conditions. Although moderate hypoxia did not change p27^kip1 ^mRNA expression, mRNA stability was slightly decreased under moderate hypoxia (Fig. 5).

The PCR products were analyzed by agarose gel electrophoresis followed by EB staining, which revealed discrete amplification products of the predicted size (Fig. 4b).

### Effects of BPS and 8-Br-cAMP on p27^kip1 ^protein expression during hypoxia

**Figure 6:**
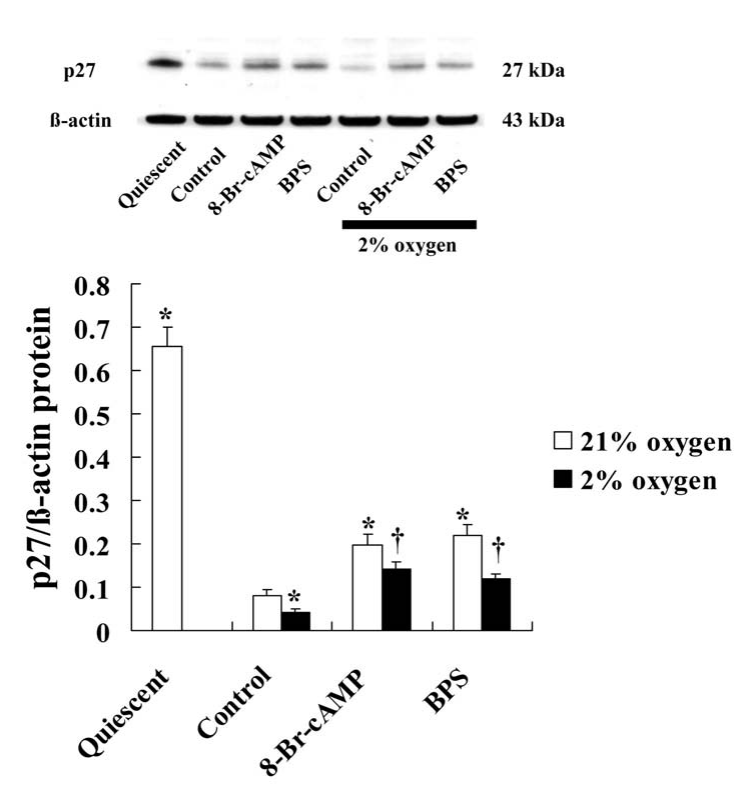
**Effects of BPS and 8-Br-cAMP on p27^kip1 ^protein expression during hypoxia**. Cultured HPASMC were exposed to 21% and 2% oxygen with or without 1 mM of 8-Br-cAMP or 10 μM of BPS for 24 hours, and then Western blotted. Hypoxia decreased p27^kip1 ^protein expression. BPS and 8-Br-cAMP each significantly increased p27^kip1 ^protein expression under both normoxia and hypoxia. Photomicrographs are representative of 4 similar experiments, and bar graphs show density ratios of p27^kip1 ^protein versus those of β-actin bands. Open and solid bars, 21% and 2% oxygen, respectively. Data are expressed as means ± SE (n = 4). *P < 0.05 versus 21% oxygen control. ^†^P < 0.05 versus 2% oxygen.

A large amount of p27^kip1 ^protein was expressed during the quiescent state after serum depletion. Serum stimulation significantly decreased p27^kip1 ^protein expression, which was significantly augmented by moderate hypoxia for 24 h. In contrast, BPS significantly blocked the reduction in p27^kip1 ^protein. Incubation with 1 mM 8-Br-cAMP and BPS similarly affected p27^kip1 ^protein expression under both normoxic and hypoxic conditions (Fig. 6).

**Figure 7:**
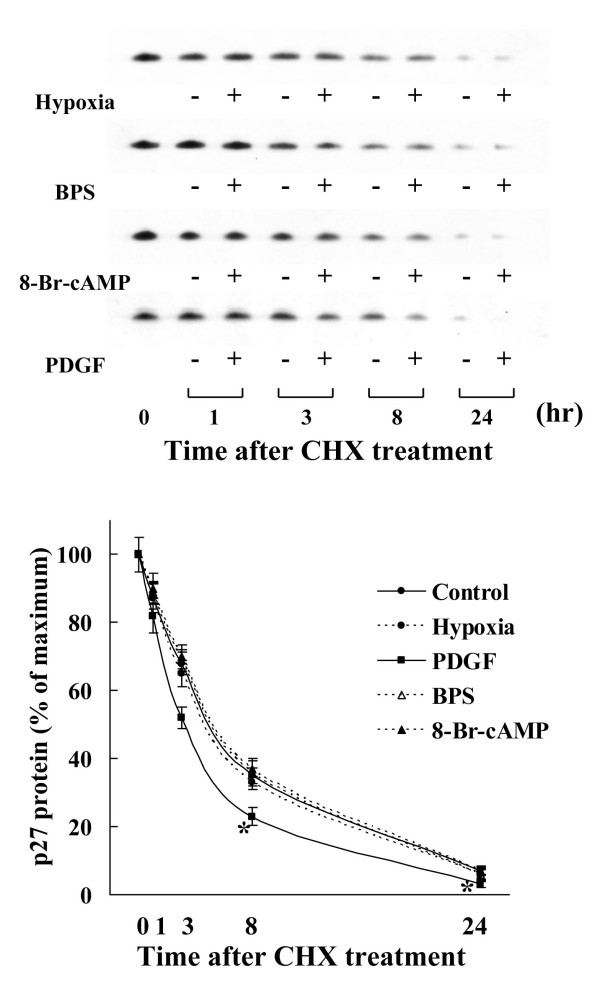
**Effects of BPS, 8-Br-cAMP, hypoxia, and PDGF on p27^kip1 ^protein stability**. Cultured HPASMC were exposed to 21% or 2% oxygen with or without 10 μM BPS, 1 mM 8-Br-cAMP, or 25 ng/ml PDGF and 25 μg/ml of CHX for indicated periods and Western blotted. Degradation of p27^kip1 ^expression did not significantly change among cells exposed to hypoxia, BPS, or 8-Br-cAMP. PDGF promoted degradation of p27^kip1 ^protein expression. Graphs show % of maximal p27^kip1 ^protein expression. Line with solid circles, 21% oxygen (control); dotted line with open circles, 2% oxygen (hypoxia); line with solid squares, PDGF; dotted line with open triangles, BPS; dotted line with solid triangles, 8-Br-cAMP. Data are expressed as means ± SE (n = 4). *P < 0.05 versus 21% oxygen.

To further understand role of hypoxia and BPS on p27^kip1 ^protein expression, we analyzed the stability of p27^kip1 ^protein using CHX. Neither BPS nor 8-Br-cAMP altered p27^kip1 ^protein stability. Moderate hypoxia did not affect the stability of p27^kip1 ^expression, but decreased the amount of p27^kip1 ^protein. We examined the effect of hypoxia-induced growth factors using PDGF, a key growth factor induced by hypoxia, on p27^kip1 ^protein stability. Under normoxic conditions, 25 ng/ml of PDGF significantly decreased p27^kip1 ^protein stability compared with the control (Fig. 7).

### Effects of hypoxia and BPS on p27^kip1 ^knockdown HPASMC proliferation

**Figure 8:**
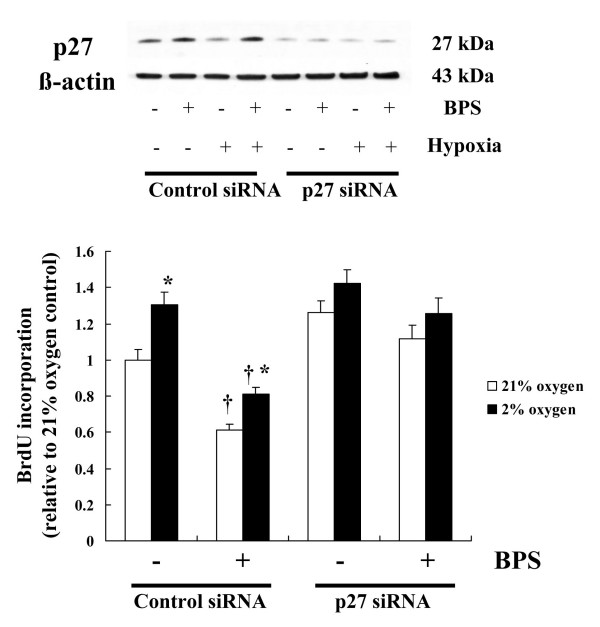
**Effects of hypoxia and BPS on BrdU incorporation in cells transfected with p27^kip1 ^siRNA**. HPASMC were transfected with control or p27^kip1 ^siRNA, then exposed to 21% and 2% oxygen with or without 10 μM BPS for 24 h. Western blot analysis showed that p27^kip1 ^protein expression in cells transfected with p27^kip1 ^siRNA was significantly suppressed under all conditions. Photomicrographs are representative of 4 similar experiments. Transfection with p27^kip1 ^siRNA significantly prevented BPS-induced inhibition of DNA synthesis. Bar graphs show BrdU incorporation relative to 21% oxygen control. Open and solid bars, 21% and 2% oxygen, respectively. Data are expressed as means ± SE (n = 6). *P < 0.05 versus with 21% oxygen; ^†^P < 0.05 versus without BPS.

To understand the role of p27^kip1 ^in terms of the inhibitory effect of BPS on cell proliferation, we examined DNA synthesis in HPASMC transfected with p27^kip1 ^siRNA under hypoxia in the presence of 10 μM BPS. Western blots showed that transfection with p27^kip1 ^siRNA significantly suppressed p27^kip1 ^protein expression. Transfection with control siRNA, which has a random sequence, did not affect p27^kip1 ^protein expression. We found that BPS increased p27^kip1 ^protein expression and significantly suppressed DNA synthesis in cells transfected with control siRNA. In contrast, transfection with p27^kip1 ^siRNA significantly decreased p27^kip1 ^protein expression and prevented the BPS-induced inhibition of DNA synthesis. In addition, moderate hypoxia significantly promoted DNA synthesis and reduced p27^kip1 ^protein expression in the control cells, but not in the cells transfected with p27^kip1 ^siRNA (Fig. 8).

## Discussion

We showed here that moderate hypoxia (2% oxygen) enhanced the proliferation of serum-stimulated HPASMC in accordance with promoted p27^kip1 ^protein degradation, probably *via *the induction of growth factors such as PDGF. We also demonstrated that BPS suppressed HPASMC proliferation under both hypoxic and normoxic conditions by blocking p27^kip1 ^mRNA degradation through an increase in intracellular cAMP. In addition, we confirmed using p27^kip1 ^gene silencing that p27^kip1 ^regulation in fact reflects HPASMC proliferation.

Increased levels of growth factors derived from the accumulation of hypoxia-inducible factor 1α (HIF-1α) are thought to regulate PASMC proliferation under hypoxic conditions since a partial HIF-1α deficiency decreases muscularizartion of pulmonary arterioles in animals exposed to chronic hypoxia [7]. Although HIF-1α regulates various transcriptional genes for angiogenic factors, severe hypoxia and iron depletion induce cell growth arrest. Our finding that severe hypoxia (0.1% oxygen) suppressed nucleotide synthesis is in line with those of others who incubated several tumor cell lines under hypoxic conditions or with iron chelators [25,29]. In contrast to severe hypoxia, other studies have indicated that moderate hypoxia (1 – 5% oxygen) enhances the proliferation of rat and bovine PASMC, airway-smooth muscle cells, lung fibroblasts and mesangial cells [30-33]. Our findings that DNA synthesis was increased during moderate hypoxia, and that the HPASMC cell cycle progresses more quickly under hypoxic than normoxic conditions were also compatible with previous findings.

The suppressive effect of hypoxia on p27^kip1 ^expression has been demonstrated in mice with pulmonary hypertension induced by hypoxia [8]. However, the expression of p27^kip1^, which blocks the cell cycle at the G_0/1 _phase, is regulated *via *several mechanisms including transcription, protein degradation and translation [34-36]. The data presented here indicated that hypoxia minimally promoted p27^kip1 ^mRNA degradation. Our data also suggested that the hypoxia-induced down-regulation of p27^kip1 ^was not apparently mediated by hypoxia *per se*, but rather mitogenic factors such as PDGF derived via hypoxia enhanced p27^kip1 ^protein degradation. We demonstrated that the decrease of p27^kip1 ^expression during hypoxia was post-transcriptional regulation from the results of RT-PCR and western blot analysis. We hypothesized that the discrepancy between the results of p27^kip1 ^protein expression and protein stability during hypoxia may be explained by the effect of CHX which could suppress the protein expression of the hypoxic signal transduction including hypoxia-induced growth factors, such as PDGF. Our results that PDGF decreased the stability of p27^kip1 ^is consistent with our hypothesis, and we believe that these results are consistent with conclusion that p27^kip1 ^down-regulation mediates hypoxia-induced HPASMC proliferation. The suppressive effect of PDGF on p27^kip1 ^expression has been demonstrated using rat aortic vascular smooth muscle cells [37] and in human saphenous vein smooth muscle cells [38], and oncogenic Ras induces cell cycle progression and shortens the half-life of p27^kip1 ^protein [39]. Since both Ras and PDGF activate mitogen-activated protein kinase (MAPK), we believe that MAPK activated through growth factors derived from hypoxia and HIF-1α enhanced the degradation induced by hypoxia.

Our results showed that HPASMC incubated with BPS were arrested at the G_0/1 _phase even under hypoxia, with p27^kip1 ^elevation being associated with increased intracellular cAMP expression, which was not affected by the oxygen concentration. These results indicated that the BPS-cAMP pathway functioned even under hypoxic conditions and that p27^kip1 ^elevation might be a consequence of BPS-induced intracellular cAMP elevation. To confirm this hypothesis, we investigated the effects of the cAMP analogue 8-Br-cAMP on p27^kip1 ^expression and of BPS on DNA synthesis in p27^kip1 ^gene knockdown HPASMC. The effects of 8-Br-cAMP and BPS on p27^kip1 ^expression were similar and p27^kip1^-dependent regulation of proliferation was confirmed in the p27^kip1 ^knockdown cells. Overexpression of p27^kip1 ^in rat PASMC decreased thymidine uptake and cellular proliferation while p27^kip1 ^knock-out PASMC from mouse had increased cellular proliferation compared with p27^kip1 ^wild-type PASMC [22]. As well, Yu et al. demonstrated that hypoxia decreased p27^kip1 ^expression in the lung and the anti-proliferative effects of heparin during hypoxia were absent in p27^kip1 ^knock-out mouse compared with p27^kip1 ^wild-type mouse [8,23]. Using p27^kip1 ^siRNA, we demonstrated that the anti-proliferative effects of BPS during hypoxia were lessened in the decrease of p27^kip1^. Therefore, we consider that our results from 27^kip1 ^siRNA experiments are consistent with the published results, and we believe that our results demonstrate the importance of p27^kip1 ^in the hypoxic regulation of PASMC proliferation and hypoxia-induced pulmonary hypertension and remodeling, which would add an important additional advancement in this field.

We also found that BPS and 8-Br-cAMP suppressed p27^kip1 ^mRNA degradation under both normoxic and hypoxic conditions. Although cAMP regulates the expression of several genes, and the control of the mRNA degradation rate by cAMP is also an important regulatory mechanism of gene expression [40-42], the mechanisms responsible for cAMP-regulated mRNA stability are not as well understood as those of transcriptional regulation. Recent findings have suggested that p27^kip1 ^mRNA stability is controlled by interactions between MAPK-dependent regulation [43] and Rho-dependent translation [44]. In addition, cAMP induces cell relaxation through Rho GTPase activation [45,46], which might be an important target of hypoxic pulmonary vascular remodeling [47,48]. These reports imply that the Rho and MAPK interaction contributes to p27^kip1 ^mRNA stability during exposure to agents that elevate cAMP and hypoxia. Therefore, to clarify the detailed mechanisms of hypoxia and cAMP with respect to p27^kip1 ^expression, additional studies are required to explain the relationship between cAMP and Rho.

## Conclusion

In summary, we found that BPS and hypoxia play critical roles in HPASMC growth through p27^kip1^-cAMP and hypoxia-induced pathways. We believe that clarification of the precise mechanisms of pulmonary smooth muscle proliferation will lead to improved therapeutic strategies that targets hypoxic pulmonary hypertension and remodelling of the pulmonary circulation.

## Competing interests

The author(s) declare that they have no competing interests.

## Authors' contributions

MK carried out the laboratory measurement and data analysis.

SM conceived the study idea and participated in the laboratory measurement and drafted the manuscript.

YD, SA, and IM participated in the design of the study.

TI supervised the study and was involved in the manuscript writing.

All authors read and approved the final manuscript.

## References

[B1] Jeffery TK, Wanstall JC (2001). Pulmonary vascular remodelling in hypoxic rats: effects of amlodipine, alone and with perindopril. Eur J Pharmacol.

[B2] Rabinovitch M, Gamble W, Nadas AS, Miettinen OS, Reid L (1979). Rat pulmonary circulation after chronic hypoxia: hemodynamic and structural features. Am J Physiol.

[B3] Rabinovitch M, Konstam MA, Gamble WJ, Papanicolaou N, Aronovitz MJ, Treves S, Reid L (1983). Changes in pulmonary blood flow affect vascular response to chronic hypoxia in rats. Circ Res.

[B4] Biernacki W, Flenley DC, Muir AL, MacNee W (1988). Pulmonary hypertension and right ventricular function in patients with COPD. Chest.

[B5] Part One MacNee W (1994). Pathophysiology of cor pulmonale in chronic obstructive pulmonary disease. Am J Respir Crit Care Med.

[B6] Semmens M, Reid L (1974). Pulmonary arterial muscularity and right ventricular hypertrophy in chronic bronchitis and emphysema. Br J Dis Ches.

[B7] Yu AY, Shimoda LA, Iyer NV, Huso DL, Sun X, McWilliams R, Beaty T, Sham JS, Wiener CM, Sylvester JT, Semenza GL (1999). Impaired physiological responses to chronic hypoxia in mice partially deficient for hypoxia-inducible factor 1alpha. J Clin Invest.

[B8] Yu L, Quinn DA, Garg HG, Hales CA (2005). Cyclin-dependent kinase inhibitor p27^kip1^, but not p21^WAF1/Cip1^, is required for inhibition of hypoxia-induced pulmonary hypertension and remodeling by heparin in mice. Circ Res.

[B9] Cooper AL, Beasley D (1999). Hypoxia stimulates proliferation and interleukin-1alpha production in human vascular smooth muscle cells. Am J Physiol.

[B10] Frid MG, Aldashev AA, Dempsey EC, Stenmark KR (1997). Smooth muscle cells isolated from discrete compartments of the mature vascular media exhibit unique phenotypes and distinct growth capabilities. Circ Res.

[B11] Lu SY, Wang DS, Zhu MZ, Zhang QH, Hu YZ, Pei JM (2005). Inhibition of hypoxia-induced proliferation and collagen synthesis by vasonatrin peptide in cultured rat pulmonary artery smooth muscle cells. Life Sci.

[B12] Schultz K, Fanburg BL, Beasley D (2006). Hypoxia and hypoxia-inducible facter-1α promote growth facter-induced proliferation of human vascular smooth muscle cells. Am J Physiol Heart Circ Physiol.

[B13] Badesch DB, McLaughlin VV, Delcroix M, Vizza CD, Olschewski H, Sitbon O, Barst RJ (2004). Prostanoid therapy for pulmonary arterial hypertension. J Am Coll Cardiol.

[B14] Clapp LH, Finney P, Turcato S, Tran S, Rubin LJ, Tinker A (2002). Differential effects of stable prostacyclin analogs on smooth muscle proliferation and cyclic AMP generation in human pulmonary artery. Am J Respir Cell Mol Biol.

[B15] Geraci MW, Gao B, Shepherd DC, Moore MD, Westcott JY, Fagan KA, Alger LA, Tuder RM, Voelkel NF (1999). Pulmonary prostacyclin synthase overexpression in transgenic mice protects against development of hypoxic pulmonary hypertension. J Clin Invest.

[B16] Nishio S, Kurumatani H (2001). Pharmacological and clinical properties of beraprost sodium, orally active prostacyclin analogue. Nippon Yakurigaku Zasshi.

[B17] Galie N, Humbert M, Vachiery JL, Vizza CD, Kneussl M, Manes A, Sitbon O, Torbicki A, Delcroix M, Naeije R, Hoeper M, Chaouat A, Morand S, Besse B, Simonneau G, Arterial Pulmonary Hypertension and Beraprost European (ALPHABET) Study Group (2002). Effects of beraprost sodium, an oral prostacyclin analogue, in patients with pulmonary arterial hypertension: a randomized, double-blind, placebo-controlled trial. J Am Coll Cardiol.

[B18] Lievre M, Morand S, Besse B, Fiessinger JN, Boissel JP (2000). Oral Beraprost sodium, a prostaglandin I2 analogue, for intermittent claudication: a double-blind, randomized, multicenter controlled trial. Circulation.

[B19] Kainoh M, Maruyama I, Nishio S, Nakadate T (1991). Enhancement by beraprost sodium, a stable analogue of prostacyclin, in thrombomodulin expression on membrane surface of cultured vascular endothelial cells via increase in cyclic AMP level. Biochem Pharmacol.

[B20] Hatakeyama M, Weinberg RA (1995). The role of RB in cell cycle control. Prog Cell Cycle Res.

[B21] Fasciano S, Patel RC, Handy I, Patel CV (2005). Regulation of vascular smooth muscle proliferation by heparin: inhibition of cyclin-dependent kinase 2 activity by p27^kip1^. J Biol Chem.

[B22] Fouty BW, Grimison B, Fagan KA, Le Cras TD, Harral JW, Hoedt-Miller M, Sclafani RA, Rodman DM (2001). p27^kip1 ^is important in modulating pulmonary artery smooth muscle cell proliferation. Am J Respir Cell Mol Biol.

[B23] Yu L, Quinn DA, Garg HG, Hales CA (2006). Gene expression of cyclin-dependent kinase inhibitors and effect of heparin on their expression in mice with hypoxia-induced pulmonary hypertension. Biochem Biophys Res Commun.

[B24] Ii M, Hoshiga M, Fukui R, Negoro N, Nakakoji T, Nishiguchi F, Kohbayashi E, Ishihara T, Hanafusa T (2001). Beraprost sodium regulates cell cycle in vascular smooth muscle cells through cAMP signaling by preventing down-regulation of p27^kip1^. Cardiovasc Res.

[B25] Gardner LB, Li Q, Park MS, Flanagan WM, Semenza GL, Dang CV (2001). Hypoxia inhibits G_1_/S transition through regulation of p27 expression. J Biol Chem.

[B26] Graff P, Amellem O, Seim J, Stokke T, Pettersen EO (2005). The role of p27 in controlling the oxygen-dependent checkpoint of mammalian cells in late G1. Anticancer Res.

[B27] Wang G, Reisdorph R, Clark RE, Miskimins R, Lindahl R, Miskimins WK (2003). Cyclin dependent kinase inhibitor p27^kip1 ^is upregulated by hypoxia via an ARNT dependent pathway. J Cell Biochem.

[B28] Mizuno S, Kadowaki M, Demura Y, Ameshima S, Miyamori I, Ishizaki T (2004). p42/44 Mitogen-activated protein kinase regulated by p53 and nitric oxide in human pulmonary arterial smooth muscle cells. Am J Respir Cell Mol Biol.

[B29] Fukuchi K, Tomoyasu S, Watanabe H, Tsuruoka N, Gomi K (1997). G_1 _accumulation caused by iron deprivation with deferoxamine does not accompany change of pRB status in ML-1 cells. Biochim Biophys Acta.

[B30] Cogo A, Napolitano G, Michoud MC, Barbon DR, Ward M, Martin JG (2003). Effects of hypoxia on rat airway smooth muscle cell proliferation. J Appl Physiol.

[B31] Krick S, Hanze J, Eul B, Savai R, Seay U, Grimminger F, Lohmeyer J, Klepetko W, Seeger W, Rose F (2005). Hypoxia-driven proliferation of human pulmonary artery fibroblasts: cross-talk between HIF-1alpha and an autocrine angiotensin system. FASEB J.

[B32] Norman JT, Clark IM, Garcia PL (2000). Hypoxia promotes fibrogenesis in human renal fibroblasts. Kidney Int.

[B33] Sahai A, Mei C, Pattison TA, Tannen RL (1997). Chronic hypoxia induces proliferation of cultured mesangial cells: role of calcium and protein kinase C. Am J Physiol.

[B34] Eto I (2006). Nutritional and chemopreventive anti-cancer agents up-regulate expression of p27^kip1^, a cyclin-dependent kinase inhibitor, in mouse JB6 epidermal and human MCF7, MDA-MB-321 and AU565 breast cancer cells. Cancer Cell Int.

[B35] Loda M, Cukor B, Tam SW, Lavin P, Fiorentino M, Draetta GF, Jessup JM, Pagano M (1997). Increased proteasome-dependent degradation of the cyclin-dependent kinase inhibitor p27 in aggressive colorectal carcinomas. Nat Med.

[B36] Philipp-Staheli J, Kim KH, Liggitt D, Gurley KE, Longton G, Kemp CJ (2004). Distinct roles for p53, p27^kip1^, and p21^Cip1 ^during tumor development. Oncogene.

[B37] Servant MJ, Coulombe P, Turgeon B, Meloche S (2000). Differential regulation of p27^kip1 ^expression by mitogenic and hypertrophic factors: Involvement of transcriptional and posttranscriptional mechanisms. J Cell Biol.

[B38] Marra DE, Simoncini T, Liao JK (2000). Inhibition of vascular smooth muscle cell proliferation by sodium salicylate mediated by upregulation of p21^Waf1 ^and p27^kip1^. Circulation.

[B39] Kawada M, Yamagoe S, Murakami Y, Suzuki K, Mizuno S, Uehara Y (1997). Induction of p27^kip1 ^degradation and anchorage independence by Ras through the MAP kinase signaling pathway. Oncogene.

[B40] Dong Y, Aronsson M, Gustafsson JA, Okret S (1989). The mechanism of cAMP-induced glucocorticoid receptor expression. Correlation to cellular glucocorticoid response. J Biol Chem.

[B41] Jungmann RA, Kelley DC, Miles MF, Milkowski DM (1983). Cyclic AMP regulation of lactate dehydrogenase. Isoproterenol and N6,O2-dibutyryl cyclic amp increase the rate of transcription and change the stability of lactate dehydrogenase a subunit messenger RNA in rat C6 glioma cells. J Biol Chem.

[B42] Sakaue M, Hoffman BB (1991). cAMP regulates transcription of the alpha 2A adrenergic receptor gene in HT-29 cells. J Biol Chem.

[B43] Sakakibara K, Kubota K, Worku B, Ryer EJ, Miller JP, Koff A, Kent KC, Liu B (2005). PDGF-BB regulates p27 expression through ERK-dependent RNA turn-over in vascular smooth muscle cells. J Biol Chem.

[B44] Vidal A, Millard SS, Miller JP, Koff A (2002). Rho activity can alter the translation of p27 mRNA and is important for RasV12-induced transformation in a manner dependent on p27 status. J Biol Chem.

[B45] Dong JM, Leung T, Manser E, Lim L (1998). cAMP-induced morphological changes are counteracted by the activated RhoA small GTPase and the Rho kinase ROKalpha. J Biol Chem.

[B46] Laufs U, Marra D, Node K, Liao JK (1999). 3-Hydroxy-3-methylglutaryl-CoA reductase inhibitors attenuate vascular smooth muscle proliferation by preventing rho GTPase-induced down-regulation of p27^kip1^. J Biol Chem.

[B47] Fagan KA, Oka M, Bauer NR, Gebb SA, Ivy DD, Morris KG, McMurtry IF (2004). Attenuation of acute hypoxic pulmonary vasoconstriction and hypoxic pulmonary hypertension in mice by inhibition of Rho-kinase. Am J Physiol Lung Cell Mol Physiol.

[B48] Hyvelin JM, Howell K, Nichol A, Costello CM, Preston RJ, McLoughlin P (2005). Inhibition of Rho-kinase attenuates hypoxia-induced angiogenesis in the pulmonary circulation. Circ Res.

